# Dietary acrylamide exposure in F344 rats and colon tumor-bearing nude *nu/nu* mice: Dataset of gene expression of cancer pathway targets and methylation status of tumor suppressor genes in colon mucosae and tumors

**DOI:** 10.1016/j.dib.2019.104763

**Published:** 2019-11-07

**Authors:** Jennifer Roberts, Rekha Mehta, Ivan Curran, Jayadev Raju

**Affiliations:** Regulatory Toxicology Research Division, Bureau of Chemical Safety, Food Directorate, Health Products and Food Branch, Health Canada, Ottawa, Ontario, Canada

**Keywords:** Acrylamide, Cancer pathway genes, Colon tumorigenesis, DNA methylation, F344 rats, nude (*nu/nu*) mice, Tumor suppressor genes, Human colon tumor xenografts

## Abstract

Dietary acrylamide, a thermally induced food contaminant, at a level (2 mg/kg diet) typifying higher occurrence in certain food products - is neither an independent carcinogen nor a tumor promoter in the colon. This is evidenced by our previous studies using the medium-term azoxymethane (AOM)-induced colon tumorigenesis assay in F344 rats and the human colon tumor xenograft model in athymic nude (*nu/nu*) mice (https://doi.org/10.1371/journal.pone.0073916) [1]. In addition, we found that acrylamide may act as a colon co-carcinogen in association with a known carcinogen (AOM) in F344 rats. Furthermore, exposure to acrylamide at 2 mg/kg in the diet was not associated with any toxicologically relevant changes in clinical biochemistry, hematology, and apical endpoints in healthy rats (exposed only to saline injections) (https://doi.org/10.1016/j.toxrep.2016.08.010) [2]. Here we report data from our previous investigation [1] on gene expression of cancer pathway targets as well as the methylation status of select tumor suppressor genes. Briefly, mRNA and DNA were extracted from (a) colon mucosae and tumors from F344 rats exposed to AOM or saline and (b) athymic nude (*nu/nu*) mice bearing human colon tumor xenografts, both exposed to dietary acrylamide at concentrations of 0 or 2 mg/kg diet for 20 and 4 weeks, respectively. RT^2^ Profiler PCR Cancer PathwayFinder Arrays (Qiagen) and EpiTect Methyl II DNA Restriction kits and PCR Assays (Qiagen) were used to detect cancer-relevant gene expression (84 genes representing 9 pathways) and the methylation status of the CpG islands associated with 22 tumor suppressor genes in colon mucosae, tumors and xenografts. Additionally, RT^2^ Profiler PCR Arrays (Qiagen) for cell cycle regulation, growth factors, inflammatory cytokines and receptors, and inflammatory response and autoimmunity were used to investigate the gene expression (84 genes in each array) of targets involved in these select cellular pathways in the colon mucosae from AOM-treated F344 rats.

**Table:** Specifications Table

Subject area	*Toxicology*
More specific subject area	*Food safety, dietary acrylamide, cancer pathway Q-PCR analysis, tumor suppressor gene methylation*
Type of data	*Figures, Tables*
How data was acquired	*All data was acquired using the Applied Biosystems 7500 Fast PCR System, including a melt curve dissociation step*
Data format	*Analyzed data, raw gene expression and methylation data (*Supplementary Files*)*
Experimental factors	*mRNA and DNA was extracted from (a) colon mucosae and tumors from rats co-exposed to dietary acrylamide and azoxymethane or saline, (b) athymic nude (nu/nu) mice bearing human colon tumor xenografts. Quantitative real-time polymerase chain reaction (Q-PCR) was performed to investigate the gene expression of several cancer pathway targets as well as the methylation status of tumor suppressor genes.*
Experimental features	*Two animal studies were conducted in (a) male F344 rats were exposed to 2 weekly* 15 mg/kg *injections of azoxymethane (AOM) or saline, and (b) male athymic nude (nu/nu) mice bearing HT-29 human colon adenocarcinoma cells-derived tumor xenografts. For the purpose of our investigations, dietary groups exposed to American Institute of Nutrition-93G basal diet containing 0 or* 2 mg/kg *diet acrylamide for 20 or 4 weeks to F344 rats or athymic nude (nu/nu) mice, respectively, were selected. Colon mucosae, tumors and xenografts were collected at necropsy and flash frozen for later nucleic acid extractions.*
Data source location	*Ottawa, Ontario, Canada*
Data accessibility	*Data is with this article*

**Table:** 

**Value of the Data** • The role of subchronic dietary exposure to food-borne acrylamide in modulating target gene expression and methylation status was assessed in colon mucosae, tumors and human tumor xenografts in two rodent models of colon tumorigenesis. • This toxicogenomic and methylation status data will add evidence to support findings of our previous colon tumorigenesis study of acrylamide exposure at dietary concentrations reflecting higher occurrence levels in certain human foods. These data of individual genomic markers and tumor suppressor gene methylation status are to be interpreted with the clinical biochemistry, hematology, pathology and colon tumor data previously reported [1,2]. • Expression profiles are provided in the form of raw Ct values that can be further processed by researchers using their own bioinformatics algorithms and analyzed with their own data. • Our data will be beneficial in updating the existing toxicity information available on food-borne acrylamide.

## 1 Data

• The dataset includes analysis of cancer pathway gene expression and methylation status of tumor suppressor gene (TSG) promoters in rat colon mucosae and tumor samples from F344 rats and human colon tumor xenografts from nude (*nu/nu*) mice, exposed to control diet or dietary acrylamide (2 mg/kg diet).
• Raw data of gene expression and methylation status of TSG promoters in the form of Ct values are available as Supplementary Data.
• Summary of significant fold changes in pathway gene expression between control- and acrylamide-fed animals is presented in Table 2, Table 3, Table 4. Table 2 presents gene expression changes for each tissue type, for each pathway tested. Table 3 presents changes in cancer pathway gene expression of each tissue type/treatment combination with respect to saline-treated control diet colon mucosae. Table 4 is a pairwise comparison for cancer pathway gene expression profiles between all rat tissue types and treatment conditions.

**Table 1:** Manufacturer (Qiagen) catalogue numbers of commercial PCR arrays used.

RT2 Profiler PCR Array	Catalogue #
Rat Cancer PathwayFinder	PARN-033Z
Human Cancer PathwayFinder	PAHS-033Z
Rat Cell Cycle	PARN-020Z
Rat Growth Factors	PARN-041Z
Rat Inflammatory Cytokines & Receptors	PARN-011Z
Rat Inflammatory Response & Autoimmunity	PARN-077Z

**Table 2:** Summary of gene expression changes between animals fed Control (no acrylamide) and Treated (2 mg/kg acrylamide) diets in each of the panels tested (*n = 6*/group). NS indicates there are no significant changes (*p* > 0.05) that are greater than 1.5-fold relative to Control.

RT2 PCR Array	Control vs acrylamide (2 mg/kg)
Saline F344rat colon mucosae	
Cancer PathwayFinder	NS
AOM F344 rat colon mucosae	
Cancer PathwayFinder	NS
Cell cycle^a^	NS
Growth factors^a^	NS
Inflammatory cytokines and receptors^a^	NS
Inflammation and autoimmune response^a^	NS
AOM F344 rat colon tumors	
Cancer PathwayFinder	NS
HT-29 colon tumor xenografts in nude (nu/nu) mouse	
Cancer PathwayFinder^a^	NS

a Data has not been provided as a Figure.

**Table 3:**
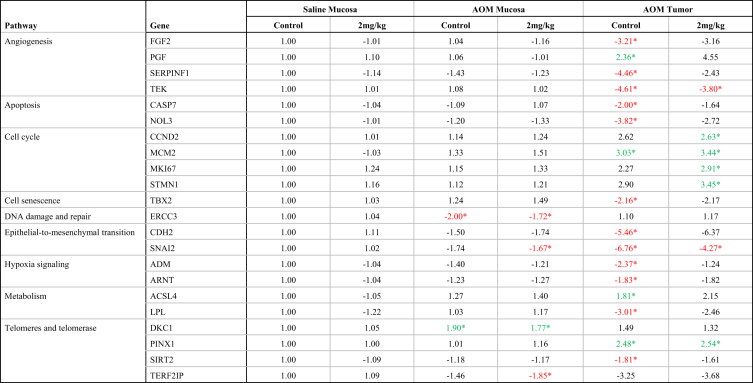
Gene expression fold changes from RT2 Cancer PathwayFinder array between Saline- and AOM-treated rats fed Control (no acrylamide) or Treated (2 mg/kg acrylamide) diets in colon mucosae and tumor tissues. (*n* = 6/group). “*” indicates significant change (*p* < 0.05) than 1.5-fold relative to the Control/Saline Mucosa group. Green = overexpressed, red = underexpressed.

**Table 4:**
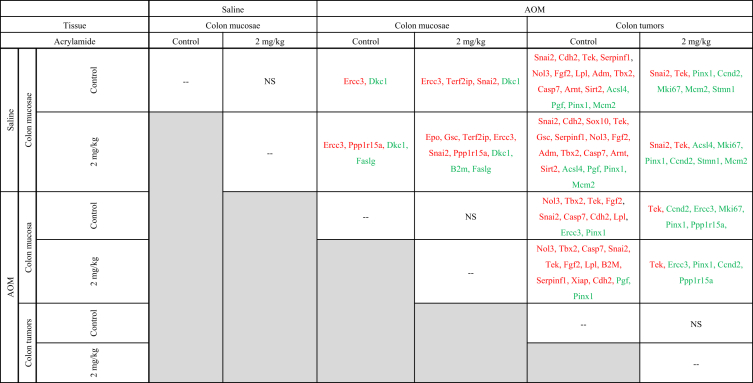
Pairwise multiple comparisons of gene expression changes from RT2 Cancer PathwayFinder array between Saline- and AOM-treated rats fed Control (no acrylamide) or Treated (2 mg/kg acrylamide) diets in colon mucosae and tumor tissues. (*n* = 6/group). NS indicates no significant changes. List of genes significantly (*p* < 0.05) greater (green) or lesser (red) than 1.5-fold relative to the Control/Saline Mucosa group, between selected pairs. Green = overexpressed, red = underexpressed.
• Summary of significant changes in TSG methylation status between control- and acrylamide-fed animals is presented in Table 5, Table 6, as well as Fig. 1, Fig. 2, Fig. 3, Fig. 4, Fig. 5, Fig. 6. Table 5 presents changes in TSG methylation status for each tissue type. Table 6 is a pairwise comparison between all rat tissue types and treatment conditions. Fig. 1 presents changes in TSG methylation status for saline-treated rat colon mucosae. Fig. 2 presents changes in TSG methylation status for AOM-treated rat colon mucosae. Fig. 3 presents a combined data comparison of TSG methylation status-related changes in saline- and AOM-treated rat colon mucosae. Fig. 4 presents changes in TSG methylation status for AOM-treated rat colon tumors. Fig. 5, **panels** A–H to present the changes in TSG methylation status for saline- and AOM-treated rat colon mucosae, and AOM-treated rat colon tumors. Fig. 6 presents changes in TSG methylation status for human colon tumour xenografts from nude (*nu/nu*) mice.

**Table 5:** Summary of tumor suppressor gene (TSG) promoter region methylation status changes between animals fed Control (no acrylamide) or Treated (2 mg/kg acrylamide) diets in each of the panels tested (*n* = 6/group). NS indicates no significant changes (*p* > 0.05) relative to Control.

Tissue	Control v. Acrylamide 2mg/kg
Saline rat colon mucosae	NS
AOM rat colon mucosae	NS
AOM rat colon tumors	NS
HT-29 colon tumor xenografts in nude (nu/nu) mouse	NS

**Table 6:**
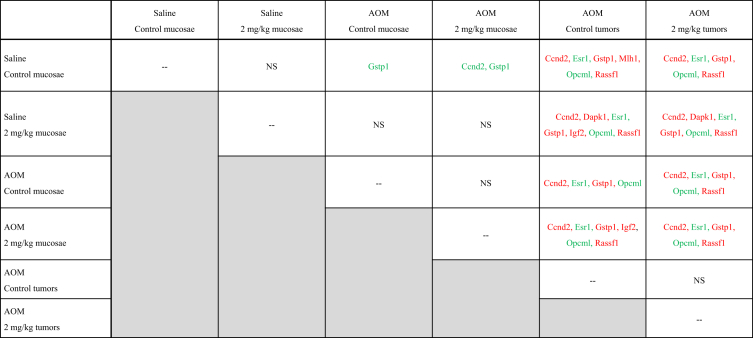
Pairwise multiple comparisons of methylation status changes between Saline- and AOM-treated rats fed Control (no acrylamide) or Treated (2 mg/kg acrylamide) diets, in colon mucosae and tumor tissues. (*n* = 6/group). NS indicates no significant changes (*p* > 0.05) between selected pairs. Green = overexpressed, red = underexpressed.

**Fig. 1:**
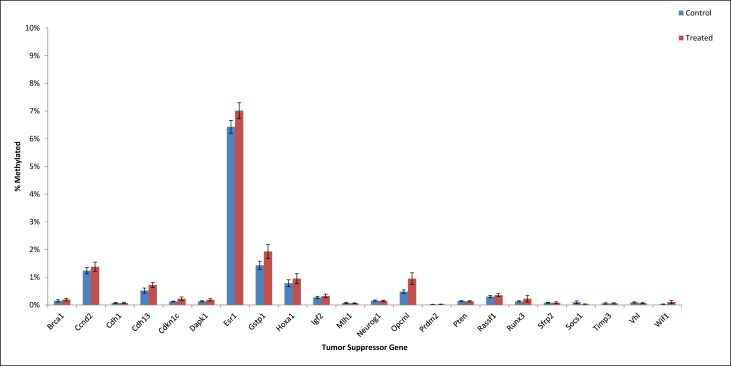
Tumor suppressor gene (TSG) promoter region methylation status in colon mucosae from Saline-treated rats fed Control (no acrylamide) or Treated (2 mg/kg acrylamide) diets, *n* = 6/group. Bars represent mean values ± SEM.

**Fig. 2:**
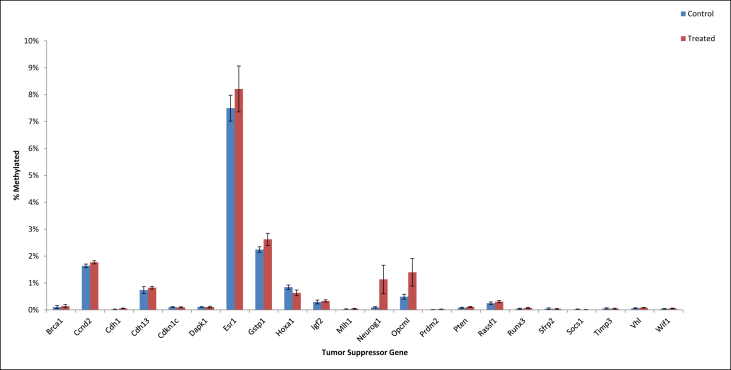
Tumor suppressor gene (TSG) promoter region methylation status in colon mucosae from AOM-treated rats fed Control (no acrylamide) or Treated (2 mg/kg acrylamide) diets, *n* = 6/group. Bars represent mean values ± SEM.

**Fig. 3:**
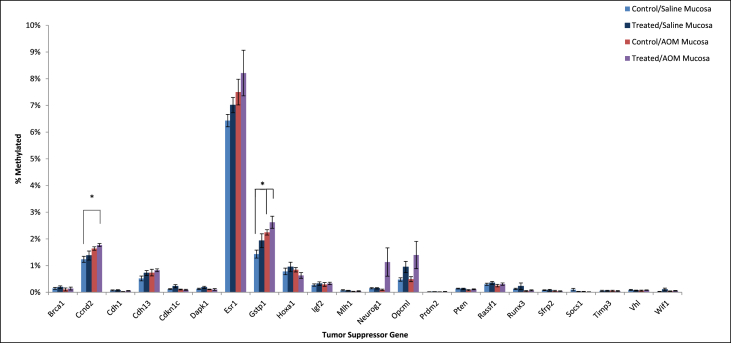
Tumor suppressor gene (TSG) promoter region methylation status in colon mucosae from Saline- and AOM-treated rats fed Control (no acrylamide) or Treated (2 mg/kg acrylamide) diets, *n* = 6/group. Bars represent mean values ± SEM. “*” indicates significant difference at *p* < 0.05 relative to Control/Saline mucosa.

**Fig. 4:**
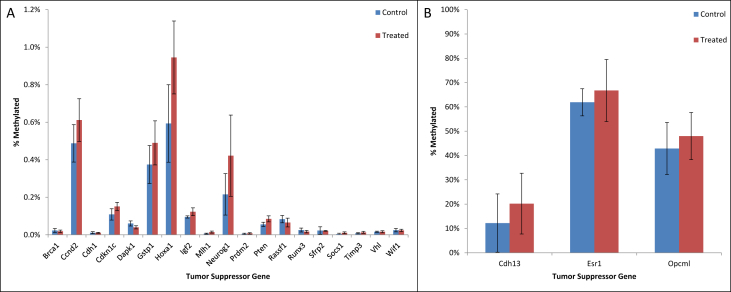
Tumor suppressor gene (TSG) promoter region methylation status in colon tumors from AOM-treated rats fed Control (no acrylamide) or Treated (2 mg/kg acrylamide) diets, *n* = 6/group. Panel (A) represent genes with low methylation status (<10%), and panel (B) represent genes with high methylation status (>10%). Bars represent mean values ± SEM.

**Fig. 5:**
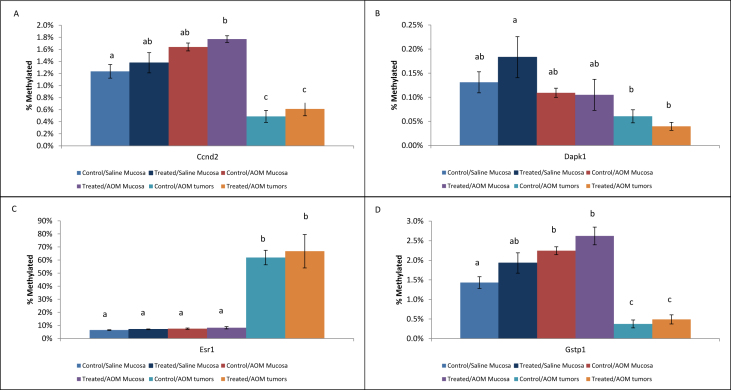

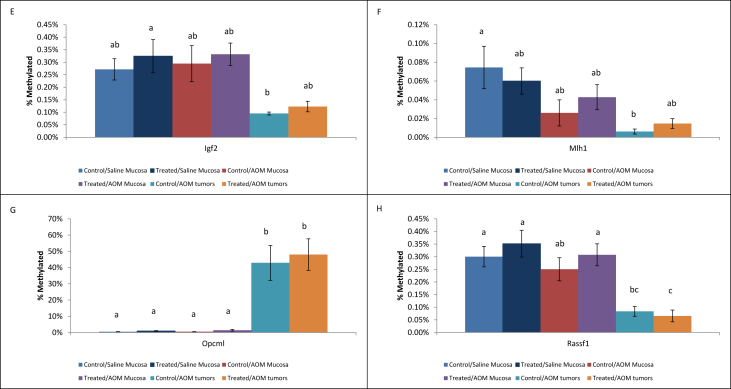
Panels (A)–(H) each represent a different tumor suppressor gene (TSG) promoter region methylation status in colon mucosae from Saline- and AOM-treated rats and colon tumors from AOM-treated rats fed Control (no acrylamide) or Treated (2 mg/kg acrylamide) diets, *n* = 6/group. Within each Panel, bars represent mean values ± SEM; and values with different letters “a”, “b”, and “c” are significantly different from each other at *p <* 0.05.

**Fig. 6:**
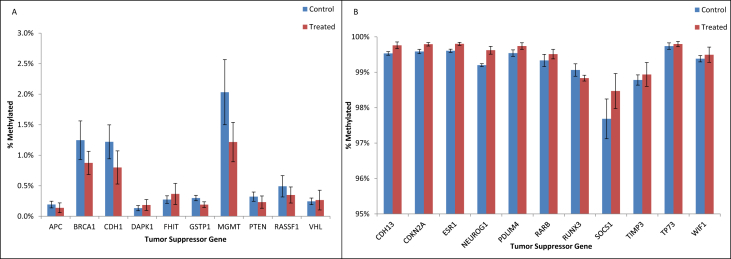
Tumor suppressor gene (TSG) promoter region methylation status in HT-29 colon tumor xenografts on athymic (*nu/nu*) mice fed Control (no acrylamide) or Treated (2 mg/kg acrylamide) diets, *n* = 6/group. Panel (A) represent genes with low methylation status (<10%), and panel (B) represent genes with high methylation status (>10%). Bars represent mean values ± SEM.

## 2 Experimental design, materials and methods

### 2.1 Animals, care and diets

The experimental protocol involving animals was reviewed and approved by the Health Canada Ottawa Animal Care Committee (ACC No. 2010-015) prior to commencement. Animals were cared for according to the guidelines of the Canadian Council on Animal Care. Male F344 rats (7 wk old) and nude (*nu/nu*) mice (6 wk old) were procured from Charles River Laboratories Canada (St. Constant, Quebec, Canada) and were pair-housed in laboratory conditions with a 12 h light/12 h dark cycle. Nude mice were housed in a Level-II isolation facility and maintained under sterile conditions. Temperature and relative humidity were controlled at 22 °C and 55%, respectively. All animals were acclimatized to the above conditions for 1 week until initiation of the experiment. The animals had free access to either lab chow (during the acclimatization phase) or experimental diets and drinking water *ad libitum*. The experimental diets were isocaloric and based on the AIN-93G rodent semisynthetic diet formula [3]. Diets were obtained from Research Diets, Inc. (New Brunswick, NJ, USA) in the form of powder. Acrylamide was mixed with the diets at the required dose using a Hobart mixer, and then made into pellets using a pelleting press. Diets were never exposed to high temperature during processing and were stored in the dark at 4 °C until use. Animals were monitored every day and their body weights and food consumption were recorded twice a week; diets were replenished weekly.

### 2.2 Experimental design

#### 2.2.1 F344 rat study

After the acclimatization phase, male F344 rats (7 wk old; *n* = 128) were randomized into four dietary groups (acrylamide at 0, 0.5, 1, 2 mg/kg diet). After 2 weeks, rats within each diet group were sub-divided to receive sub-cutaneous injections of either AOM (15 mg/kg BW; *n* = 24 rats/diet group) or saline (0.2 mL/rat; *n* = 8 rats/diet group) All animals remained on the experimental diets for 20 weeks post AOM/saline injections, after which they were sacrificed. Colons were dissected, flushed with ice-cold PBS, and slit open on a cold plate. Visible tumors were excised, the colon mucosa was divided into proximal (caecal) and distal (anal) halves and was scraped with glass slides, and both were snap-frozen in RNA*later* stabilizing agent (Invitrogen, California, USA) in liquid N_2_ for molecular analysis.

#### 2.2.2 Nude mouse study

Male athymic nude (*nu/nu*) mice (6 wk old, *n* = 48), housed in Level II containment under sterile conditions, were injected subcutaneously in the flank with HT-29 human colon adenocarcinoma cells (2 × 10^6^). After 3 weeks, mice were randomized into 4 dietary groups (acrylamide at 0, 0.5, 1, 2 mg/kg diet; *n* = 12 per diet) and tumor xenografts were measured twice a week. After 4 weeks on diet, the mice were killed and the tumors excised and snap frozen in liquid N_2_.

### 2.3 Gene expression analysis

For the purpose of this investigation, tissues from animals that were exposed to control (no acrylamide) and 2 mg/kg acrylamide were utilized. RNA was extracted from the rat distal colon mucosae (AOM and saline cohorts), rat colon tumors (AOM cohorts) and human colon tumor xenografts (nude mouse study) using RNEasy Lipid kits (cat.# 74804, Qiagen, Hilden, Germany). RT^2^ Profiler PCR Cancer PathwayFinder Arrays (Qiagen) for human and rat enabled gene expressions analysis of 84 genes representative of 9 different biological pathways involved in transformation and tumorigenesis (Table 1). In addition, RT^2^ Profiler PCR Arrays for cell cycle regulation, growth factors, inflammatory cytokines and receptors, and inflammatory response and autoimmunity were used to investigate the gene expression of targets involved in these processes in rat distal colon mucosae from AOM-treated animals only. Gene expression levels were normalized against the geometric mean of top 5 reference gene candidates (scored using geNorm software). For data reporting, only mean values with a fold change of 1.5 or greater (including those statistically significant at *p* < 0.05) are reported.

### 2.4 Epigenetics analysis

For the purpose of this investigation, control animals and the 2 mg/kg diet acrylamide groups were utilized. DNA was extracted from rat distal colon mucosae (AOM and saline cohorts), rat colon tumors (AOM cohorts) and human colon tumor xenografts (nude mouse study) using DNEasy Blood and Tissue kits (cat.# 69504, Qiagen) with an RNase A treatment (cat.# 19101, Qiagen). EpiTect Methyl II DNA Restriction Enzyme kits (cat.# 335452, Qiagen) and Methyl II PCR Array Tumor Suppressor Genes, Signature Panels (cat.# EARN-551Z, rat; cat.# EAHS-551Z, human; Qiagen) were used to detect the methylation status of the CpG islands associated with 22 tumor suppressor genes (TSG). Methylation status is presented as the percentage of DNA that is methylated or un-methylated relative to the total input DNA for each gene of interest; the methylated fraction represents genomic DNA that contained two or more methylated CpG sites in the targeted region of a gene.

### 2.5 Statistical analysis

Data was analyzed performed using SigmaPlot 12.0. Statistical comparisons were performed using a one-way ANOVA test with pairwise multiple comparisons using the Holm-Sidak method. For all tests, *p* < 0.05 was considered as statistically significant.
